# African Horse Sickness Virus Serotype 1 on Horse Farm, Thailand, 2020

**DOI:** 10.3201/eid2708.210004

**Published:** 2021-08

**Authors:** Napawan Bunpapong, Kamonpan Charoenkul, Chanakarn Nasamran, Ekkapat Chamsai, Kitikhun Udom, Supanat Boonyapisitsopa, Rachod Tantilertcharoen, Sawang Kesdangsakonwut, Navapon Techakriengkrai, Sanipa Suradhat, Roongroje Thanawongnuwech, Alongkorn Amonsin

**Affiliations:** Chulalongkorn University, Bangkok, Thailand

**Keywords:** African horse sickness, AHS, emergence, horses, outbreak, vector-borne infections, viruses, Thailand

## Abstract

To investigate an outbreak of African horse sickness (AHS) on a horse farm in northeastern Thailand, we used whole-genome sequencing to detect and characterize the virus. The viruses belonged to serotype 1 and contained unique amino acids (95V,166S, 660I in virus capsid protein 2), suggesting a single virus introduction to Thailand.

African horse sickness virus (AHSV) is an RNA virus of the family *Reoviridae*, genus *Orbivirus*. AHSV can be classified into 9 serotypes according to virus capsid protein (VP) 2 (*1*). Serotypes 1–8 have been reported from restricted areas of sub-Saharan Africa only. Serotype 9 is more widespread and causes epidemics outside Africa. Serotype 4 caused outbreaks in Spain and Portugal during 1987–1990 (*2*). 

In Thailand, the first AHS outbreak was reported in March 2020 in northeastern Thailand (*3*–*5*). AHS outbreaks have been reported in 17 provinces of Thailand, affecting ≈2,700 horses (Appendix Table 1) (*6*). We report a comprehensive outbreak investigation of emerging AHSV and whole-genome characterization of AHSV recovered from a horse farm in northeastern Thailand.

## The Study

In March 2020, the Veterinary Diagnostic Laboratory at Chulalongkorn University (Bangkok, Thailand) was notified of unusual horse deaths on a recreational horse farm, which encompasses up to 6,400 m^2^, in Nakhon Ratchasima Province, northeastern Thailand. A total of 49 horses (2 thoroughbred, 21 miniature, 26 native horses) were kept on free range. Other animals on the farm were 3 dogs, 3 rabbits, 3 pigs, and 8 peacocks. The outbreak investigation and sample collection were conducted under the approval of Institutional Animal Care and Use Committee protocol no. 2031050.

**Figure 1:**
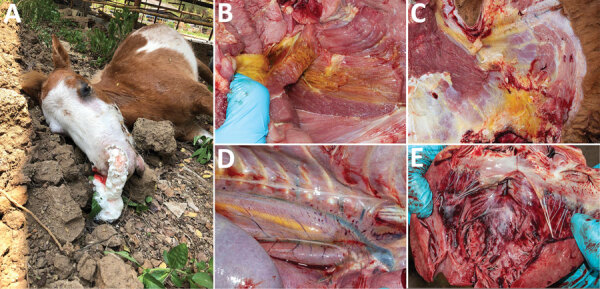
Gross lesions from horses affected by African horse sickness, Thailand, 2020. A) Mild edema at the supraorbital fossa with frothy exudate from the nostrils; B) yellow, gelatinous infiltrations and perineural edema of the intramuscular tissues; C) right axillary subcutaneous edema; D) periaortic edema and hemorrhage; E) subendocardial petechiae and ecchymoses of the heart.

On March 20, 2020, the outbreak began when horses showed severe clinical signs including depression, fever, dyspnea, and subcutaneous edema in the temporal or supraorbital area, followed by sudden death within 48 hours. On March 28, we visited the horse farm, implemented insect-proof housing, and collected a blood sample from a horse with clinical signs (horse CU-1), which died the next day. We performed necropsies on 2 horse carcasses (CU-2 and CU-3) and collected 7 tissue samples. Gross lesions showed frothy exudate in the bronchial lumen and mild edema of the supraorbital sinus and conjunctiva. We observed intermuscular and perineural edema at the axillary region and subcutaneous muscle, periaortic edema, and subendocardial hemorrhage (Figure 1). Histopathologic slides showed congestion of the spleen, liver, lymph nodes, and lung; no other remarkable lesions were observed. The outbreak lasted 3 weeks and affected 30 horses (last case on April 10). On April 26, horses on the farm were vaccinated with polyvalent, live-attenuated AHSV vaccine (Ondersterpoort Biological Products, https://www.obpvaccines.co.za); no horses showed clinical signs after vaccination and implementation of insect-proof housing. In total, during the 3 weeks of the outbreak, the mortality rate for horses on the farm was 61.22% (30 deaths/49 horses) (Appendix Table 2). Mortality rates by breed were 100% (2/2) for thoroughbreds, 76.19% (16/21) for miniature horses, and 46.15% (12/26) for native horses. The same management practices were applied for horses of all breeds. 

We visited the horse farm again on May 30 (1 month after vaccination) and August 1 (3 months after vaccination). From the remaining horses we collected 18 serum samples at each visit (total 36). All samples were tested for antibodies against AHSV by blocking ELISA specific to VP7 (INgezim AHSV Compac Plus; Eurofins Technologies, https://ingenasa.eurofins-technologies.com) (Appendix). All 36 serum samples were positive for AHSV antibodies (Appendix Table 3).

To identify AHSV, we extracted viral RNA from 8 blood and tissue samples by using the GeneAll GENTi Viral DNA/RNA Extraction Kit (GeneAll, http://www.geneall.com). We performed real-time reverse transcription PCR (RT-PCR) with VP7 gene–specific primers and probes by using the SuperScript III Platinum One-Step qRT-PCR System (Thermo Fisher, https://www.thermofisher.com) (Appendix) (*7*). All 8 samples were positive for AHSV (cycle threshold 28.29–33.91). In detail, blood samples from horse CU-1; lymph nodes from CU-2; and lymph node, lung, spleen, heart, liver, and kidney samples from CU-3 were positive for AHSV (Appendix Table 4). To further characterize AHSV from Thailand, we performed VP2 gene-specific RT-PCR, which showed that the AHSVs from Thailand belong to AHSV serotype 1 (*8*). We next subjected the spleen from horse CU-3 to whole-genome sequencing and 2 additional viruses (from CU-1 and CU-2) to VP2 and nonstructural gene (NS) 3 gene sequencing (Table). We conducted whole-genome sequencing by amplifying viral fragments and sequencing by using MinION Oxford Nanopore technologies (https://nanoporetech.com) (Appendix Table 5) (*9*). The nucleotide sequences of the AHSVs from Thailand were submitted to GenBank (accession nos. MW387422–35). Nucleotide sequences of AHSV from Thailand were pairwise compared against those of vaccine and reference viruses. We found that the whole genome of Thailand AHSV (virus CU-3) possessed high nucleotide identities (99.40%–100%) to the reference Thailand AHSV-1 (110983/63 and TAI2020/01). For the VP2 gene, Thailand AHSV possessed 99.90% nucleotide identities among them; the highest nucleotide identity (99.90%) was to the reference Thailand AHSV-1 (110983/63 and TAI2020/01, 02, and 03). The nucleotide identities of VP2 between Thailand AHSV and the reference AHSV of serotypes 2–9 were low (54.60%–67.10%). For the NS3 gene, Thailand AHSV had 99.90% nucleotide identities; the highest nucleotide identity was to the reference South Africa AHSV of clade gamma (97.10%–99.90%) (Appendix Table 6).

**Table:** Characterization of African horse sickness virus isolated during study of African horse sickness on horse farm, Thailand, 2020*

Virus	Host horse				Nucleotide sequences, GenBank accession nos.		
	Sex	Age	Breed		WGS	VP2	NS3
CU-1	F	3	Miniature		NA	MW387422	MW387423
CU-2	F	3	Miniature		NA	MW387424	MW387425
CU-3	F	2	Miniature		MW387426–35	MW387427	MW387435

*NA, not available; NS, nonstructural gene; VP, viral capsid protein; WGS, whole-genome sequences (10 segments).

**Figure 2:**
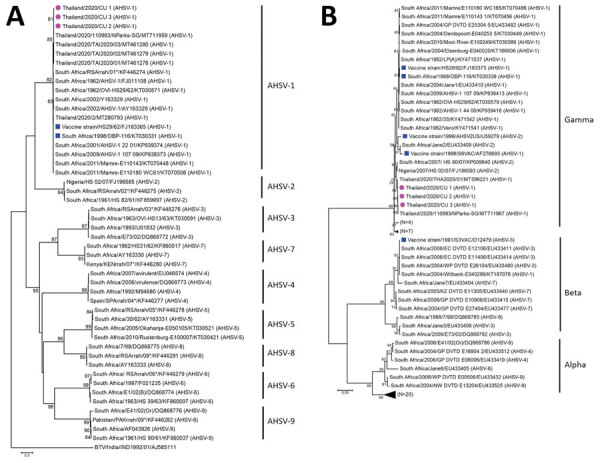
Phylogenetic trees for AHSV, Thailand, 2020. A) Viral capsid protein 2; B) nonstructural gene 3. Purple circles indicate Thailand AHSV characterized in this study; blue squares indicate AHSV vaccine strains; numbers after AHSV indicate serotypes. Scale bars indicate nucleotide substitutions per site. AHSV, African horse sickness virus.

For phylogenetic analysis, we included the VP2 sequences of the Thailand AHSV and reference viruses (AHSV-1 vaccine strains and AHSV serotypes 1–9). For phylogenetic analysis of NS3, we included the NS3 sequences of Thailand AHSV and reference viruses of alpha, beta, and gamma clades. The maximum clade credibility trees for VP2 and NS3 genes were constructed by using BEAST 2.0 (https://beast.community) with the Bayesian Markov chain Monte Carlo algorithm (Appendix). Phylogenetic analysis of the VP2 gene showed that Thailand AHSV was clustered in AHSV serotype 1 but not in other clusters (serotypes 2–9). For NS3, the Thailand AHSVs were grouped within the gamma clade, similar to the references AHSV-1 and AHSV-2 (Figure 2). We analyzed amino acid determinants of VP2 and NS3 at 2 neutralizing epitopes (residues 321–339 and 377–400) (*10*). Thailand AHSV had identical amino acids at positions 321–339 and 377–400 among Thailand AHSVs and some reference AHSV-1 but differed from the reference vaccine strains (HS29/62 and OBP-116). The deduced amino acids related to the virulence of AHSV at positions 357 of VP2 and 165–168 and 201 of NS3 were also analyzed (*1*,*11*). Thailand AHSV contained virulence-related amino acids at VP2–357N and NS3–201M, which were not observed in some reference AHSV-1 and AHSV vaccines (Appendix Table 7). Of note, all Thailand AHSVs contained unique amino acids at positions 95V, 166S, and 660I, suggesting a single introduction from the same AHSV ancestor into Thailand.

## Conclusions

We speculate that AHSV serotype 1 potentially spread outside Africa from imported subclinically infected animals, such as zebras. The Thailand government implemented control measures to prevent further spread, including movement restrictions, quarantine, disinfection, and vector control. Moreover, to prevent spread in Thailand and neighboring countries, mass vaccination of equids with a live-attenuated AHSV vaccine was conducted. The AHSV from Thailand possessed unique amino acids, suggesting a single introduction of the virus to the country. This information will be useful for strategic planning for disease prevention and control, vaccine selection, and diagnostic assay development.

Appendix Supplemental methods and results for study of African horse sickness virus serotype 1 on horse farm, Thailand, 2020.
