# Supplementary material for: African Horse Sickness Virus Serotype 1 on Horse Farm, Thailand, 2020
Source: Emerg Infect Dis. 2021 Aug;27(8):2208–11. doi: 10.3201/eid2708.210004 (PMC8314833; doi:10.3201/eid2708.210004)
Supplement: Appendix — Supplemental methods and results for study of African horse sickness virus serotype 1 on horse farm, Thailand, 2020. [file 21-0004-Techapp-s1.pdf]

Article DOI: https://doi.org/10.3201/eid2708.210004

African Horse Sickness Virus Serotype 1 on Horse Farm, Thailand, 2020

Appendix

African Horse Sickness virus outbreak investigation and sample collection

In March 2020, the Veterinary Diagnostic Laboratory, Chulalongkorn University, was notified of an unusual death of horses in a horse farm located in Nakhon Ratchasima province, northeastern Thailand. On 28 March 2020, we visited the horse farm and collected blood sample (n=1) from a horse with clinical signs (CU-1), which then reported death on the next day. We also performed necropsy on 2 horse carcasses (CU-2 and CU-3) and collected tissue samples (n=7). All samples were tested for AHSV by real-time RT-PCR specific to VP7. On 26 April 2020, the farm implemented polyvalent, live-attenuated AHS vaccination. After vaccination, we visited the horse farm on 30 May 2020 and 1 August 2020 (1 month and 3 months after vaccination), and 36 serum samples (n=18 each visit) were collected from the remaining horses. All samples were tested for antibodies against AHSV by blocking ELISA specific to VP7. Outbreak investigation and sample collection were conducted under the approval of the faculty of Veterinary Science, Chulalongkorn University, Animal Care and Usage Protocol (IACUC# 2031050).

African Horse Sickness virus identification

To identify AHSV, viral RNA was extracted from blood and tissue samples (n=8) by using the GeneAll® GENTiTM Viral DNA/RNA Extraction Kit (GeneAll®; Lisbon, Portugal). Real-time RT-PCR with VP7 gene specific primers and probes was performed by using the SuperScriptTM III Platinum ® One-Step Quantitative RT-PCR system (InvitrogenTM, CA, USA) (*1*). In brief, one-step RT-PCR was conducted in a total final volume of 25 µl comprising 2 µl of template RNA, 1x buffer reaction mix, 2.5 µM of forward and reverse primers and 0.4 µM of probe, 1 unit of SuperScript III RT, and distilled water to reach the final volume. The real-

time RT-PCR reaction contained a cDNA synthesis step at 55°C for 30 min, followed by an initial denaturation step at 94°C for 2 min; and 45 cycles of denaturation at 94°C for 30 s, annealing at 53°C for 30 s and extension at 68°C for 30 s. AHSV serotype identification was performed using RT-PCR with VP2 gene-specific primers using a SuperScriptTM III Platinum® One-Step Quantitative RT-PCR System (InvitrogenTM; California, USA) (*2*). In brief, one-step RT-PCR was conducted in a total final volume of 25 µl, comprising 2 µl of template RNA, 1x buffer reaction mix, 2.5 µM of forward and reverse primers, 1 unit of SuperScript III RT, and distilled water to reach the final volume. The PCR product was detected by 1.5% agarose gel electrophoresis.

African Horse Sickness virus antibody detection

Antibodies against AHSV were tested by blocking ELISA specific to VP7 (INgezim AHSV Compac Plus, Madrid, Spain). The blocking ELISA is based on the use of a recombinant antigen (VP7 protein) and monoclonal antibody (MAb) specific to the VP7 protein of AHSV. The assay was performed according to the instruction protocol of INgezim AHSV Compac Plus, Madrid, Spain. In brief, 100 µl of a 1:5 diluted serum sample was dispensed into each well. 100 ul of positive and negative controls were added to control wells and incubated at 37°C for 1 hour. The plate was washed for 5 times, and 100 µl of conjugate was added to each well and incubated at 37°C for 30 minutes. The plate was then washed 5 times, and 100 µl of substrate was added in to each well and incubated at room temperature for 10 minutes. Then, 100 µl of stop solution was added. The OD of the sample was read with a spectrophotometer at 450 nm within 5 minutes after the addition of stop solution. The blocking percentage (BP) was calculated. Samples showing BP values <45% were considered negative, 45%-50% were considered as suspected and >50% were considered positive for AHSV antibodies.

African Horse Sickness virus characterization

To characterize Thai-AHSV, one virus (CU-3) was subjected to whole genome sequencing using MinION Oxford Nanopore technologies (Oxford, UK). Additional two viruses (CU-1 and CU-2) were subjected to VP2 and NSP3 gene sequencing by oligonucleotide primer sets previously described or new primer sets designed using the Primer 3 plus program (*3*).

Nucleotide sequences were assembled using CLC software (QIAGEN, CA, USA). The nucleotide sequences of the Thai-AHSVs were published in the GenBank database (accession # MW387422-35).

To perform pairwise comparisons, nucleotide sequences of each Thai-AHSV gene were aligned with those of vaccine and reference viruses using MEGA v10.0 (Tempe, AZ, USA) and MegAlign software v.5.03 (DNASTAR Inc.). To perform phylogenetic analysis, the VP2 nucleotide sequences of the Thai-AHSV and reference viruses, including AHSV-1 vaccine strains, AHSV serotype 1-9 and bluetongue virus (outgroup), were included in the analysis. For phylogenetic analysis of NSP3, the NSP3 sequences of Thai-AHSV and reference viruses of alpha, beta and gamma clades were included in the analysis. The maximum clade credibility (MCC) trees of VP2 and NSP3 genes were constructed by BEAST 2.0 with the Bayesian Markov-Chain Monte Carlo (BMCMC) algorithm using the BEAST 2.0 program (*4*). To perform genetic analysis of Thai-AHSV, deduced amino acids of VP2 and NSP3 were aligned using MEGA v10.0 (Tempe, AZ, USA) and MegAlign software v.5.03 (DNASTAR Inc.). Deduced amino acids of each gene of the viruses were aligned and analyzed for genetic characteristics. VP2 contains two neutralizing epitopes at residues 321-339 and 377-400 (*5*), and the deduced amino acids related to the virulence of AHSV at positions 357 of VP2 and 165-168 and 201 of NSP3 were analyzed (*6*,*7*).

References

<jrn>1. Guthrie AJ, Maclachlan NJ, Joone C, Lourens CW, Weyer CT, Quan M, et al. Diagnostic accuracy of a duplex real-time reverse transcription quantitative PCR assay for detection of African horse sickness virus. J Virol Methods. 2013;189:30–5. PubMed https://doi.org/10.1016/j.jviromet.2012.12.014</jrn>

<jrn>2. Maan NS, Maan S, Nomikou K, Belaganahalli MN, Bachanek-Bankowska K, Mertens PP. Serotype specific primers and gel-based RT-PCR assays for ‘typing’ African horse sickness virus: identification of strains from Africa. PLoS One. 2011;6:e25686. PubMed https://doi.org/10.1371/journal.pone.0025686</jrn>

<jrn>3. Quan M, van Vuuren M, Howell PG, Groenewald D, Guthrie AJ. Molecular epidemiology of the African horse sickness virus S10 gene. J Gen Virol. 2008;89:1159–68. PubMed https://doi.org/10.1099/vir.0.83502-0</jrn>

<jrn>4. Suchard MA, Lemey P, Baele G, Ayres DL, Drummond AJ, Rambaut A. Bayesian phylogenetic and phylodynamic data integration using BEAST 1.10. Virus Evol. 2018;4:vey016. PubMed https://doi.org/10.1093/ve/vey016</jrn>

<jrn>5. Martínez-Torrecuadrada JL, Langeveld JPM, Meloen RH, Casal JI. Definition of neutralizing sites on African horse sickness virus serotype 4 VP2 at the level of peptides. J Gen Virol. 2001;82:2415–24. PubMed https://doi.org/10.1099/0022-1317-82-10-2415</jrn>

<jrn>6. Potgieter AC, Wright IM, van Dijk AA. Consensus sequence of 27 African horse sickness virus genomes from viruses collected over a 76-year period (1933 to 2009). Genome Announc. 2015;3:e00921-15. PubMed https://doi.org/10.1128/genomeA.00921-15</jrn>

<jrn>7. van Staden V, Smit CC, Stoltz MA, Maree FF, Huismans H. Characterization of two African horse sickness virus nonstructural proteins, NS1 and NS3. Arch Virol Suppl. 1998;14:251–8. PubMed https://doi.org/10.1007/978-3-7091-6823-3_22</jrn>

Appendix Table 1. Summary of AHS outbreaks in Thailand reported to OIE

Reported date, dd/mm/yyyy	Outbreak	Province	Date of start of the outbreak	Total animal affected	Cases	Deaths	Apparent morbidity rate	Apparent mortality rate	Apparent case fatality rate	Proportion susceptible animals lost
27/03/2020	1	Nakhon Ratchasima	24/02/2020	1171	460	436	39.28%	37.23%	94.78%	37.23%
03/04/2020	2	Chon Buri	27/03/2020	33	6	5	18.18%	15.15%	83.33%	15.15%
03/04/2020	3	Prachuap Khiri Khan	27/03/2020	145	20	20	13.79%	13.79%	100.00%	13.79%
10/04/2020	4	Ratchaburi	01/04/2020	164	7	6	4.27%	3.66%	85.71%	3.66%
10/04/2020	5	Phetchaburi	04/04/2020	627	44	40	7.02%	6.38%	90.91%	6.38%
17/04/2020	6	Chaiyaphum	29/03/2020	3	1	1	33.33%	33.33%	100.00%	33.33%
24/04/2020	7	Sa Kaeo	14/04/2020	22	4	3	18.18%	13.64%	75.00%	13.64%
Vaccination*			19-20/04/2020							
01/05/2020	8	Saraburi	24/04/2020	281	45	34	16.01%	12.10%	75.56%	12.10%
15/05/2020	9	Lop Buri	05/05/2020	2	1	1	50.00%	50.00%	100.00%	50.00%
15/05/2020	10	Phra Nakhon Si Ayutthaya	06/05/2020	23	3	3	13.04%	13.04%	100.00%	13.04%
22/05/2020	11	Chachoengsao	13/05/2020	43	8	8	18.60%	18.60%	100.00%	18.60%
22/05/2020	12	Nakhon Nayok	18/05/2020	12	2	2	16.67%	16.67%	100.00%	16.67%
19/06/2020	13	Nonthaburi	10/06/2020	79	1	1	1.27%	1.27%	100.00%	1.27%
26/06/2020	14	Pathum Thani	14/06/2020	58	1	1	1.72%	1.72%	100.00%	1.72%
26/06/2020	15	Bangkok	16/06/2020	11	1	1	9.09%	9.09%	100.00%	9.09%
14/10/2020	16	Buri Ram	11/08/2020	7	1	1	14.29%	14.29%	100.00%	14.29%
14/10/2020	17	Prachin Buri	01/09/2020	54	2	2	3.70%	3.70%	100.00%	3.70%
				2735	607	565	22.19%	20.66%	93.08%	20.66%

*Implementation of vaccination by the Department of Livestock Development, Thailand.

Appendix Table 2. Cumulative mortality of AHS outbreaks in a horse farm investigated in this study

Date, dd/mm/yyyy	Miniature Death	Miniature Cumulative mortality	Native Death	Native Cumulative mortality	Thoroughbred Death	Thoroughbred Cumulative mortality	Total Death	Total Cumulative mortality
20/3/2020			2	2 (7.69%)			2	2 (4.08%)
22/3/2020	1	1 (4.76%)					1	3 (6.12%)
23/3/2020	1	2 (9.52%)	1	3 (11.54%)			2	5 (10.205%)
24/3/2020	1	3 (14.28%)					1	6 (12.24%)
26/3/2020	2	5 (23.81%)	1	4 (15.38%)			3	9 (18.37%)
27/3/2020	2	7 (33.33%)					2	11 (22.45%)
28/3/2020	3*	10 (47.62%)					3	14 (28.57%)
29/3/2020	1†	11 (52.38%)	3	7 (26.92%)	1	1 (50.00%)	5	19 (38.75%)
30/3/2020			1	8 (30.77%)			1	20 (40.82%)
31/3/2020	2	13 (61.90%)					2	22 (44.89%)
1/4/2020					1	2 (100.00%)	1	23 (46.93%)
2/4/2020			1	9 (34.61%)			1	24 (48.98%)
4/4/2020	1	14 (66.67%)					1	25 (51.02%)
5/4/2020	1	15 (71.43%)					1	26 (53.06%)
6/4/2020			1	10 (38.46%)			1	27 (55.10%)
8/4/2020			1	11 (42.31%)			1	28 (57.14%)
9/4/2020	1	16 (74.19%)					1	29 (59.18%)
10/4/2020			1	12 (46.15%)			1	30 (61.22%)
Total	16/21 (76.19%)		12/26 (46.15%)		2/2 (100%)		30/49 (61.22%)	

*Horses (CU-2 and CU-3) were subjected to necropsy and tissue sample collection.
†Horse (CU-1) was subjected to blood collection and was reported dead on the next day.

Appendix Table 3. Serological test for AHSV antibodies by blocking ELISA in a horse farm investigated in this study

ID*	Sex	Breed	Vaccination Date	AHSV blocking ELISA 30/05/2020	AHSV blocking ELISA 01/08/2020
CU-4	Mare	Crossed	26/04/2020	Positive	Positive
CU-5	Stallion	Mixed	26/04/2020	Positive	Positive
CU-6	Stallion	Miniature	26/04/2020	Positive	Positive
CU-7	Stallion	Miniature	26/04/2020	Positive	Positive
CU-8	Mare	Miniature	26/04/2020	Positive	Positive
CU-9	Stallion	Miniature	26/04/2020	Positive	Positive
CU-10	Mare	Miniature	26/04/2020	Positive	Positive
CU-11	Mare	Native	26/04/2020	Positive	Positive
CU-12	Filly	Miniature	26/04/2020	Positive	Positive
CU-13	Stallion	Native	26/04/2020	Positive	Positive
CU-14	Mare	Native	26/04/2020	Positive	Positive
CU-15	Colt	Native	26/04/2020	Positive	Positive
CU-16	Mare	Native	26/04/2020	Positive	Positive
CU-17	Stallion	Miniature	26/04/2020	Positive	Positive
CU-18	Stallion	Native	26/04/2020	Positive	Positive
CU-19	Gelding	Crossed	26/04/2020	Positive	Positive
CU-20	Stallion	Native	26/04/2020	Positive	Positive
CU-21	Gelding	Native	26/04/2020	Positive	Positive

*Animal ID was assigned for remaining horses with vaccination and blood sample collection in May and August, 2020.

Appendix Table 4. Description of AHSV detection from samples in this study

ID	Sex	Age	Breed	Status at time of sample collection	Clinical signs*	Real-time RT PCR† (Ct value) EDTA blood	Lymph node	Lung	Spleen	Heart	Liver	Kidney
CU-1	F	3	Miniature	Alive	Yes	+ (30.69)	N/A	N/A	N/A	N/A	N/A	N/A
CU-2	F	3	Miniature	Dead	Yes	N/A	+ (28.29)	N/A	N/A	N/A	N/A	N/A
CU-3	F	2	Miniature	Dead	Yes	N/A	+ (28.79)	+ (33.91)	+ (28.48)‡	+ (31.92)	+ (30.16)	+ (33.35)

*Clinical signs including depression, fever, dyspnea and subcutaneous edema, were presented in the temporal or supraorbital area.
†Real-time RT PCR specific to the VP7 gene,
‡WGS.

Appendix Table 5. List of primers for VP2 and NS3 sequencing of AHSV

Primer name	Sequence (5′→3′)	Position	Product size (bp)	Temp.	Reference
AHSV-OIE_qF	AGAGCTCTTGTGCTAGCAGCCT	1038-1059	79	60	(*1*)
AHSV-OIE_qR	GAACCGACGCGACACTAATGA	1096-1116			
AHSV-OIE_qProbe	FAM-TGCACGGTCACCGCT-MGB	1080-1094			
MQ.AHS.NS3.1-22F	GTTTAAATTATCCCTTGTCATG	3-22	758	45	(*3*)
MQ.AHS.NS3.749-769R	GTAAGTCGTTATCCCGGCTC	739-758			
AHSV-1- VP2_22_F	TTATTTCAGCATGGCGTCTG	1-22	1155	50	This Study
AHSV-1- VP2_1157_R	CAAAGCTTACCATTCGGATCA	1137-1157			
AHSV-1-VP2_815_F	GCCGAGATGGCTAGATCAAT	796-815	854	52	This Study
AHSV-1-VP2_1649_R	CCTCTCTCTTCCCCGACATT	1630-1649			
AHSV-1-VP2_1315_F	GGTCGTTGACACAATCATGC	1296-1315	842	52	This Study
AHSV-1-VP2_2137_R	CTTTCACTCGTTCCCCTCTG	2118-2137			
AHSV-1-VP2_1795_F	CGTTGATGATCCGCAAACTT	1776-1795	872	52	This Study
AHSV-1-VP2_2647_R	CAACCGCAATAACTCTCAAGC	2627-2647			
AHSV-1-VP2_2306_F	CCGTGAAGGATTGAGCTTTT	2232-2251	906	51	This Study
AHSV-1-VP2_3215_R	TCACACCGTTACTCTATCTTCGAC	3114-3137			

Appendix Table 6. Pairwise comparison of the whole genome sequence of Thai-AHSV and those of reference AHSV

					Gene									
Virus	Serotype*	Accession #	Clade†	Country	VP1	VP2	VP3	VP4	VP5	VP6	VP7	NS1	NS2	NS3
This study														
CU-3	AHSV-1	This study	Gamma	Thailand	100.00 (100.00)	100.00 (100.00)	100.00 (100.00)	100.00 (100.00)	100.00 (100.00)	100.00 (100.00)	100.00 (100.00)	100.00 (100.00)	100.00 (100.00)	100.00 (100.00)
CU-1	AHSV-1	This study	Gamma	Thailand	N/A	99.90 (99.90)	N/A	N/A	N/A	N/A	N/A	N/A	100.00 (100.00)	99.90 (99.90)
CU-2	AHSV-1	This study	Gamma	Thailand	N/A	99.90 (99.90)	N/A	N/A	N/A	N/A	N/A	N/A	99.80 (99.80)	99.90 (99.90)
Vaccine strains														
HS29/62	AHSV-1 (Vaccine)	FJ183365	Gamma	South Africa	88.80 (98.20)	96.40 (98.30)	94.80 (99.60)	93.50 (98.00)	84.90 (97.90)	95.90 (94.50)	95.50 (99.70)	97.40 (99.30)	96.20 (98.60)	97.60 (99.50)
OBP-116	AHSV-1 (Vaccine)	KT030331	Gamma	South Africa	88.80 (98.20)	96.40 (98.30)	94.80 (99.60)	93.50 (98.00)	84.90 (97.90)	95.90 (94.50)	95.50 (99.70)	97.40 (99.30)	96.20 (98.60)	97.60 (99.50)
Reference strains														
110983/63-Thailand-NParks-SG	AHSV-1	MT711959-967	Gamma	Thailand	100.00 (100.00)	99.90 (99.90)	99.90 (100.00)	99.90 (99.90)	100.00 (100.00)	100.00 (100.00)	100.00 (100.00)	99.90 (100.00)	99.80 (99.80)	99.40 (99.00)
TAI2020/01	AHSV-1	MT586213-221	Gamma	Thailand	100.00 (100.00)	99.90 (99.90)	100.00 (100.00)	99.80 (99.70)	100.00 (100.00)	99.90 (100.00)	100.00 (100.00)	99.90 (100.00)	100.00 (100.00)	99.80 (100.00)
TAI2020/02	AHSV-1	MT461279	Gamma	Thailand	N/A	99.90 (99.90)	N/A	N/A	N/A	N/A	N/A	N/A	N/A	N/A
TAI2020/03	AHSV-1	MT461280	Gamma	Thailand	N/A	99.90 (99.90)	N/A	N/A	N/A	N/A	N/A	N/A	N/A	N/A
Mokopane-E120203	AHSV-2	KT030360-369	Gamma	South Africa	95.50 (98.80)	67.10 (71.00)	97.00 (99.40)	99.10 (99.20)	95.90 (99.60)	98.20 (97.80)	94.70 (99.40)	97.80 (99.30)	99.20 (99.70)	97.10 (99.50)
DG25423	AHSV-3	KP939486-567	Beta	South Africa	88.80 (98.20)	56.90 (51.90)	95.20 (99.30)	93.50 (98.00)	71.80 (78.90)	95.20 (93.10)	96.40 (99.70)	97.50 (99.50)	96.20 (98.60)	28.10 (10.50)
AHSV-4 90 01	AHSV-4	KP939577-694	Alpha	South Africa	88.70 (98.00)	56.30 (49.90)	94.60 (99.40)	93.50 (98.10)	70.70 (75.40)	96.10 (94.70)	87.70 (98.60)	97.00 (99.50)	97.00 (98.90)	65.90 (67.00)
AHSV-5 93 00	AHSV-5	KP939707-797	Gamma	South Africa	98.70 (98.90)	55.10 (49.60)	97.40 (99.70)	94.30 (97.80)	71.90 (77.30)	30.70 (10.80)	94.80 (99.10)	97.70 (99.60)	96.50 (99.50)	97.40 (98.00)
AHSV-6 68 00	AHSV-6	KP939808-925	Alpha	South Africa	95.30 (98.90)	55.20 (50.10)	97.30 (99.40)	99.10 (99.40)	71.40 (79.80)	98.60 (98.60)	95.20 (100.00)	96.10 (99.30)	99.50 (99.70)	66.70 (67.50)
AHSV-7 89 09	AHSV-7	KP939934-997	Beta	South Africa	95.80 (98.90)	57.70 (53.00)	96.90 (99.60)	96.40 (98.90)	71.60 (77.90)	27.90 (8.50)	99.10 (100.00)	97.90 (99.30)	99.30 (99.70)	67.30 (67.50)
AHSV-8 9B 98	AHSV-8	KP940008-116	Alpha	South Africa	95.40 (98.90)	55.10 (49.30)	97.20 (99.70)	93.70 (97.30)	70.50 (75.00)	30.30 (10.80)	95.10 (100.00)	96.30 (98.70)	97.90 (98.90)	66.50 (67.90)
AHSV-9 6 01	AHSV-9	KP940128-235	Alpha	South Africa	95.20 (98.90)	54.60 (48.40)	97.20 (99.40)	96.70 (99.10)	71.40 (79.50)	96.90 (96.90)	95.10 (99.40)	96.20 (99.10)	96.90 (98.90)	66.50 (68.40)

*Classification based on VP2.
†Classification based on NS3.

Appendix Table 7. Genetic analysis of VP2 and NS3 genes of Thai-AHSV and reference AHSV

					VP2						NSP3		
					Unique amino acids			Neutralizing antibodies					
Virus	Host	Country	Year	Accession number	I95V	G166S	T660I	321-339*	377-400*	K357N	Accession number	K201M	165-168
Vaccine serotype1													
HS29/62	N/A	N/A	N/A	FJ183365	I	G	T	KKRKEGDDLTARNTFRQAL	MNIDPNGKLWIEHKQTVSEQLKKK	K	FJ183373	K	MLLA
OBP-116	N/A	South Africa	1988	KT030331	I	G	T	KKRKEGDDLTARNTFRQAL	MNIDPNGKLWIEHKQTVSEQLKKK	K	KT030339	K	MLLA
Reference serotype 1													
E160445 PRL160501	E.caballus	South Africa	2016	KX987209	I	G	T	KKRKEGDDLTARNAFRQAL	MNIDPNGKLWIEHKQTVSEQLKKK	N	KX987217	M	MLLA
E160445 PRL160500	E.caballus	South Africa	2016	KX987199	I	G	T	KKRKEGDDLTARNAFRQAL	MNIDPNGKLWIEHKQTVSEQLKKK	N	KX987207	M	MLLA
E160440 EP09828	E.caballus	South Africa	2016	KX987189	I	G	T	KKRKEGDDLTARNAFRQAL	MNIDPNGKLWIEHKQTVSEQLKKK	N	KX987197	M	MLLA
Onderstepoort-E060043	E.caballus	South Africa	2006	KT030501	I	G	T	KKRKEGDDLTARNTFRQAL	MNIDPNGKLWVEHKQTVSEQLKKK	N	KT030509	M	MLLA
Hartbeespoort-E050075	E.caballus	South Africa	2005	KT030471	I	G	T	KKRKEGDDLTARNTFRQAL	MNIDPNGKLWVEHKQTVSEQLKKK	N	KT030479	M	MLLA
107 09	N/A	South Africa	2009	KP939373	I	G	T	KKRKEGDDLTARNTFRQAL	MNIDPNGKLWIEHKQTVSEQLKKK	K	KP939413	K	MLLA
RSArrah/01*	N/A	South Africa	N/A	KF446274	I	G	T	KKRKEGDDLTARNTFRQAL	MNIDPNGKLWIEHKQTVSEQLKKK	K	N/A	N/A	N/A
110983/63	E.caballus	Thailand	2020	MT711959	V	S	I	KKRKEGDDLTARNTFRQAL	MNIDPNGKLWVEHKQTVSEQLKKK	N	MT711967	M	MLLA
TAI2020/01	E.caballus	Thailand	2020	MT461278	V	S	I	KKRKEGDDLTARNTFRQAL	MNIDPNGKLWVEHKQTVSEQLKKK	N	MT586221	M	MLLA
TAI2020/02	E.caballus	Thailand	2020	MT461279	V	S	I	KKRKEGDDLTARNTFRQAL	MNIDPNGKLWVEHKQTVSEQLKKK	N	N/A	N/A	N/A
TAI2020/03	E.caballus	Thailand	2020	MT461280	V	S	I	KKRKEGDDLTARNTFRQAL	MNIDPNGKLWVEHKQTVSEQLKKK	N	N/A	N/A	N/A
This study													
CU-1	E.caballus	Thailand	2020		V	S	I	KKRKEGDDLTARNTFRQAL	MNIDPNGKLWVEHKQTVSEQLKKK	N		M	MLLA
CU-2	E.caballus	Thailand	2020		V	S	I	KKRKEGDDLTARNTFRQAL	MNIDPNGKLWVEHKQTVSEQLKKK	N		M	MLLA
CU-3	E.caballus	Thailand	2020		V	S	I	KKRKEGDDLTARNTFRQAL	MNIDPNGKLWVEHKQTVSEQLKKK	N		M	MLLA

*Amino acid position based on AHS serotype 4 (EUO46574).
